# Iatrogenic T-Cell Lymphoma with Associated Hemophagocytic Lymphohistiocyotsis in a Patient with Long-Standing Rheumatoid Arthritis

**DOI:** 10.1155/2018/8097965

**Published:** 2018-01-11

**Authors:** X. A. Andrade, H. E. Fuentes, D. M. Oramas, H. Mann, P. Kovarik

**Affiliations:** ^1^Department of Medicine, John H. Stroger Jr. Hospital of Cook County, Chicago, IL, USA; ^2^Division of Pathology, University of Illinois at Chicago, Chicago, IL, USA; ^3^Department of Pathology, John H. Stroger Jr. Hospital of Cook County, Chicago, IL, USA

## Abstract

Patients with rheumatoid arthritis are at increased risk of hematological malignancies, especially when exposed to immunosuppressive therapy. The mechanisms of lymphomagenesis remain poorly understood but factors implicated include high disease activity, exposure to antitumoral necrosis factor medications, and Epstein–Barr virus infection. Lymphoid malignancies of T-cell origin are uncommon in patients with rheumatoid arthirits. Clinical presentation with associated hemophagocytic lymphohistiocyotsis is rare and confers a poor prognosis. This case report illustrates a case of a patient with long-standing rheumatoid arthritis and an iatrogenic peripheral T-cell lymphoma with secondary hemophagocytic lymphohistiocytosis who achieved a complete response after intensive chemotherapy.

## 1. Introduction

Patients with rheumatoid arthritis (RA) have an increased risk of lymphoid malignancies compared to the general population. Several factors have been associated with increased risk of a secondary malignancy including chronic immunosuppressive treatment, anti-TNF medications, high disease activity, and long-standing disease. The mechanism of lymphomagenesis behind these factors and their implications in management and surveillance of lymphoid malignancies in patients with RA remain uncertain.

T-cell malignancies in patients with RA are uncommon, representing less than 10% of all lymphomas. Although secondary hemophagocytic lymphohistiocyotsis (HLH) is a rare presentation of T-cell lymphomas, it is an independent adverse prognostic factor. Recognition of T-cell lymphoma-associated HLH is key for a prompt diagnosis and treatment and improved outcomes.

We present a case of a patient with T-cell lymphoma-associated HLH and long-standing RA years after conventional immunosuppressive and biologic treatment.

## 2. Case Presentation

The patient is a 56-year-old woman with a history of rheumatoid arthritis diagnosed 11 years prior to admission. She was treated with methotrexate, hydroxychloroquine, and etanercept with good symptomatic control. Three years prior to admission, she developed etanercept-related scleromalacia perforans on her right eye and was switched to infliximab therapy.

The patient presented with a one-week history of fever, sore throat, and abdominal pain. Physical examination was relevant for jaundice, bilateral tonsillar enlargement, cervical and axillary lymphadenopathy (2 cm in diameter), and diffuse abdominal tenderness without guarding. Her neurological examination was unremarkable.

Laboratory testing showed altered liver function tests (AST 149 UI/L, ALT 69 UI/L, alkaline phosphatase 317 UI/L, gamma GT 305 UI/L, total bilirubin 9.9 mg/dl, and direct bilirubin 6.6 mg/dl), bicytopenia (WBC 8.2 × 10^9^/L, neutrophils 58%, lymphocytes 7%, monocytes 15%, hemoglobin 10.6 g/dl, hematocrit 32%, and platelets 49.0 × 10^9^/L), hyperferritinemia (ferritin 4150 ng/ml), and hypertriglyceridemia (triglycerides 402 mg/dl). Thoracic, abdominal, and pelvic computerized tomography scans showed diffuse generalized lymphadenopathy and splenomegaly with nodular hypodensities.

Due to the suspicion of a hematological malignancy, a bone marrow aspiration and a biopsy were performed which showed hypercellularity with a nodular and interstitial infiltration by small- to intermediate-sized atypical lymphocytes, macrophages with hemophagocytosis, and an inconclusive flow cytometry result for a lymphoproliferative disorder. The bone marrow karyotype was normal.

**Figure 1:**
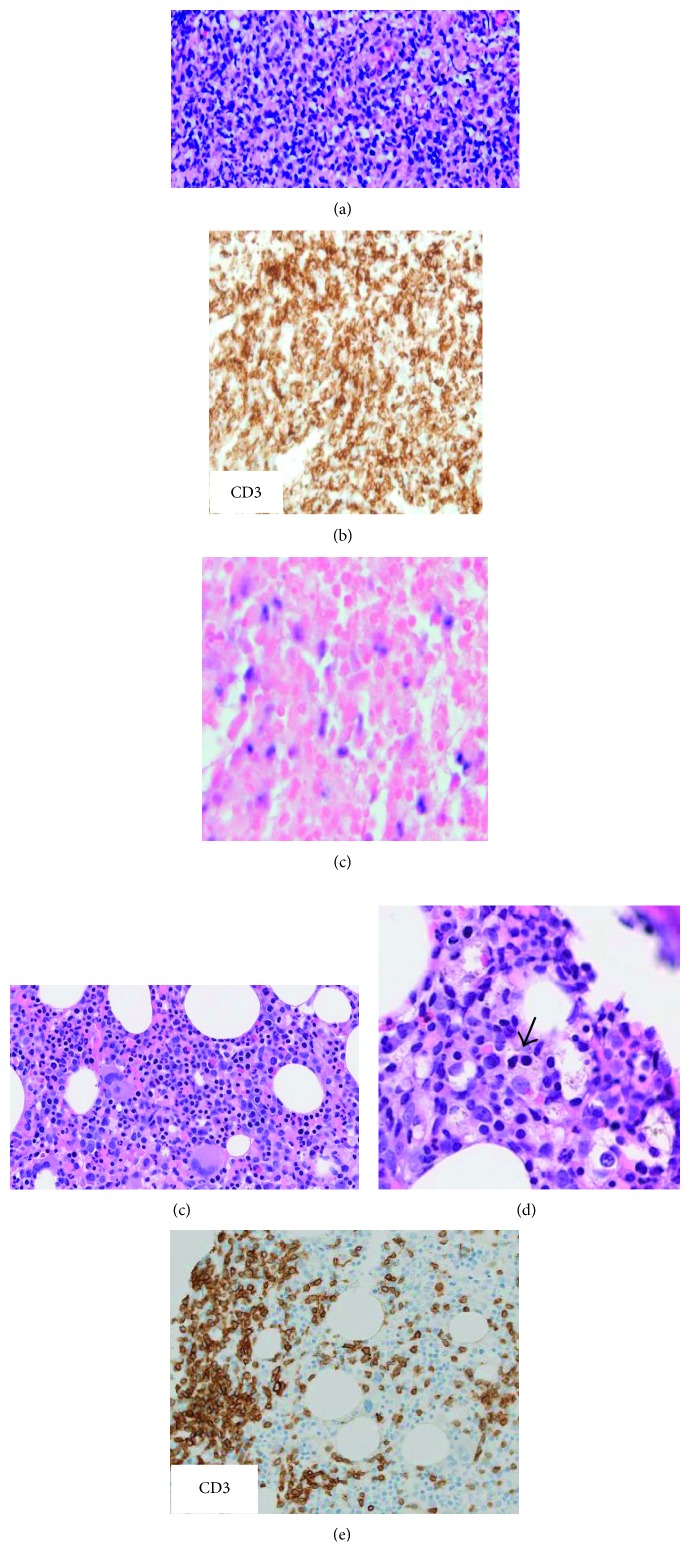
Lymph node biopsy shows small- to medium-sized atypical lymphocytes (a). In this infiltrate, immunohistochemistry is positive for CD3 (b) and in situ hybridization is positive for Epstein–Barr virus (c). Bone marrow biopsy shows clusters of atypical lymphocytes (d) and histiocytes with hemophagocytosis (e). Immunohistochemical staining for CD3 is positive (f).

An axillary lymph node core biopsy was performed which showed a lymphoid infiltrate composed of small, intermediate, and large atypical lymphoid cells with foci of necrosis and angiocentric and angioinvasive features. Immunohistochemical stains were positive for CD3, CD5, and CD7, costained both CD4 and CD8 in atypical lymphoid cells, and displayed negative staining for CD10, CD20, CD30, and CD15. In situ hybridization for Epstein–Barr virus (EBV) was positive on atypical lymphoid cells coexpressing CD4 and CD8 (Figure 1). Karyotyping of lymph node tissues was not performed. Liver biopsy showed sinusoidal, lobular, and portal infiltrates of small- to intermediate-sized atypical lymphoid cells. Immunohistochemical stains showed that these cells were positive for CD3, CD5, CD7, and both CD4 and CD8 (Figure 1).

The patient was diagnosed with an iatrogenic immunodeficiency-related T-cell lymphoma and malignancy-associated hemophagocytic lymphohistiocyotsis. Immunosuppressive therapy was discontinued at the time of diagnosis, and intravenous steroids were admistered on day 3 of admission. Ultimately, chemotherapy was started with CHOEP regimen (cyclophosphamide, doxorubicin, vincristine, etoposide, and prednisone) every 21 days. After chemotherapy cycle 3, the patient showed significant improvement of cytopenias and liver function tests (Table 1).

The patient completed 6 cycles of chemotherapy without major complications. Positron emission computerized tomography imaging after the 6th cycle demonstrated a complete metabolic response. Currently, the patient remains in complete response two years after diagnosis and continues to receive medical care in our institution.

## 3. Discussion

Many descriptive studies have reported an increased incidence of lymphomas in patients with RA compared to the general population [1, 2]. Impaired T-cell function due to high disease activity [3] and chronic immunosuppressive therapy with methotrexate [4] have been implicated in lymphomagenesis; however, this association remains unclear as results from large descriptive studies have shown conflicting results [5, 6]. More recently, anti-TNF medications received close attention due to several reports of lymphoid malignancies in patients with RA but a metanalysis by Bongartz et al. could not find convincing evidence for this association [7].

The relationship between Epstein–Barr virus (EBV) infections and lymphomagenesis is well established in patients with B-cell malignancies and chronic immunosuppression, but its role in T-cell malignancies is less known. There is a high prevalence of EBV infection in patients with T-cell lymphomas (TCLs) reaching around 25–58% [8, 9], but the mechanism of lymphomagenesis remains poorly understood. Even though there is heterogeneity in the methods of detection for EBV infection between retrospective studies, it has been consistently linked to worse overall and progression-free survival in patients with TCLs [8, 10].

Considering all lymphoid malignancies in patients with RA, TCLs represent less than 10% [4, 11]. Initial presentation as lymphoma-associated HLH (LAHLH) is more common on TCLs compared to those of B-cell origin [12]. However, the actual incidence of LAHLH in patients with RA and lymphoid malignancies is unclear and likely underreported due to the lack of standardized diagnostic criteria [13, 14].

There are no large prospective controlled studies to evaluate optimal therapy of TCLs and LAHLH. Most of the patients are treated with CHOP regimen (cyclophosphamide, adriamycin, vincristine, and prednisone), and etoposide is frequently added given its proven benefit on pediatric protocols for primary HLH [14] as in the case presented here.

TCLs with LAHLH have a significantly worse overall survival when compared with TCLs without LAHLH (2 months versus 45 months, resp.) as reported in retrospective studies [12, 15]. Multivariate analysis on a small retrospective study by Han et al. suggests that LAHLH is an independent prognostic factor associated with poor survival after adjustment for other known unfavorable features [12]. The benefit of a more intensive chemotherapy regimen and consolidation of response with bone marrow transplant is still uncertain and an open area of investigation.

## 4. Conclusion

LAHLH should be considered in patients with long-standing RA and unexplained fever, cytopenias, organomegaly, and lymphadenopathy, particularly if exposed to chronic immunosuppressive therapy and anti-TNF medications. When present, secondary HLH is an independent risk factor for poor prognosis. The benefit of a more intensive chemotherapy and consolidation with bone marrow transplant remains uncertain. Large trials with risk stratification and subgroup analysis may help elucidate this question.

The outcome of the patient presented in this case is exceptional. Arguably, early diagnosis and treatment with etoposide may have contributed to favorable outcomes.

## Figures and Tables

**Table 1:** Clinical and laboratory findings through admission and during chemotherapy.

	Day 0	Day 3	Day 7	Cycle 1	Cycle 2	Cycle 3	Cycle 4	Cycle 5	Cycle 6
WBC (×10^9^/L)	8.2	6.0	3.0	3.0	5.0	4.1	5.2	4.8	3.9
Hemoglobin (g/dl)	10.6	9.3	8.3	8.5	12.5	12.7	8.9	9.4	12.6
Platelets (×10^9^/L)	41	62	129	110	97	147	170	68	181
Ferritin (ng/ml)	4150	—	—	—	—	—	—	—	—
Triglycerides (mg/dl)	400	—	—	—	—	—	—	—	155
AST (U/L)	149	213	111	94	68	40	27	18	16
ALT (U/L)	69	107	113	111	95	34	19	16	20
LDH (U/L)	979	1,093	540	529	427	299	287	154	136
Total bilirubin (mg/dl)	9.5	14	9.3	11.2	3.3	1.2	0.6	0.5	0.6
Direct bilirubin (mg/dl)	5.8	9.2	6.8	6.8	1.3	0.5	0.3	0.2	0.3
Temperature (°Fahrenheit)	102.2	98.2	98.7	97.8	—	—	—	—	—
Heart rate (bpm)	130	134	84	75	—	—	—	—	—

Methylprednisone dosed at 1 mg/kg IV was started on admission day 3; cycle 1 of R-CHOP was started on admission day 9; cycles 1 to 3 were administered with a 50% dose reduction of adriamycin and vincristine due to abnormal liver function tests; cycles 2 to 6 included etoposide 100 mg/m^2^ IV on days 1 to 3.
